# Omega-3 Monoacylglyceride Effects on Longevity, Mitochondrial Metabolism and Oxidative Stress: Insights from *Drosophila melanogaster*

**DOI:** 10.3390/md16110453

**Published:** 2018-11-16

**Authors:** Camille M. Champigny, Robert P. J. Cormier, Chloé J. Simard, Patrick-Denis St-Coeur, Samuel Fortin, Nicolas Pichaud

**Affiliations:** 1 Department of Chemistry and Biochemistry, Université de Moncton, Moncton, NB E1A 3E9, Canada; camille.champigny@univ-tlse3.fr (C.M.C.); erc3945@umoncton.ca (R.P.J.C.); ecs0175@umoncton.ca (C.J.S.); patrick-denis.st-coeur@umoncton.ca (P.-D.S.-C.); 2 SCF Pharma, Ste-Luce, QC G0K 1P0, Canada; sfortin@scfpharma.com

**Keywords:** mitochondrial metabolism, aging, monoacylglyceride, polyunsaturated fatty acids, oxidative stress

## Abstract

During the last decade, essential polyunsaturated fatty acids (PUFAs) such as eicosatetraenoic acid (EPA) and docosahexaenoic acid (DHA) derived from marine sources have been investigated as nonpharmacological dietary supplements to improve different pathological conditions, as well as aging. The aim of this study was to determine the effects of dietary n-3 PUFA monoacylglycerides (MAG, both EPA and DHA) on the mitochondrial metabolism and oxidative stress of a short-lifespan model, *Drosophila melanogaster*, sampled at five different ages. Our results showed that diets supplemented with MAG-EPA and MAG-DHA increased median lifespan by 14.6% and decreased mitochondrial proton leak resulting in an increase of mitochondrial coupling. The flies fed on MAG-EPA also had higher electron transport system capacity and mitochondrial oxidative capacities. Moreover, both n-3 PUFAs delayed the occurrence of lipid peroxidation but only flies fed the MAG-EPA diet showed maintenance of superoxide dismutase activity during aging. Our study therefore highlights the potential of n-3 PUFA monoacylglycerides as nutraceutical compounds to delay the onset of senescence by acting directly or indirectly on the mitochondrial metabolism and suggests that Drosophila could be a relevant model for the study of the fundamental mechanisms linking the effects of n-3 PUFAs to aging.

## 1. Introduction

Essential polyunsaturated fatty acids (PUFAs) are crucial components of human nutrition as they cannot be synthesized endogenously by human cells. Among these fatty acids, the omega-3 family (n-3 PUFA) including eicosatetraenoic acid (EPA, 20:5n-3) and docosahexaenoic acid (DHA, 22:6n-3) has been shown to be an important determinant of the structure and function of mammalian cells [1,2]. EPA and DHA are abundant in algae and marine animals, which represent a major source for these PUFAs. Different formulations of omega-3 supplements are now available such as ethyl esters, triacylglycerides, free fatty acids, phospholipids and monoacylglycerides which allow these compounds to be tested as sustainable dietary supplements. Another source of omega-3 for humans comes from the conversion of a shorter chain omega-3 fatty acid, α-linolenic acid, (ALA, 18:3n-3) that can be found in many commonly eaten plants [2,3,4].

ALA can be converted to EPA, which is further transformed to DHA through the sequential action of several enzymes such as elongases, as well as Δ^6^-desaturase and Δ^5^-desaturase [3,4]. Interestingly, retroconversion of DHA to EPA via peroxisomal and/or mitochondrial oxidation has also been demonstrated in different models [5,6,7]. The conversion efficiency from ALA is however rather low for both EPA and DHA [8,9]. EPA and DHA are consequently favored as omega-3 dietary supplements and the last decade has seen a surge of studies that tested these omega-3 as nonpharmacological dietary supplements to improve different pathological conditions such as inflammation, autoimmune diseases as well as cardiovascular and brain disorders [10,11,12,13].

More recently, these omega-3 supplements have also been associated with the health status of organisms, promoting protection of several tissues against aging [10,14,15,16], although some studies on mice gave contradictory results [17,18]. A prospective cohort study also recently showed that higher levels of circulating omega-3 from seafood, especially EPA, is associated with a higher likelihood of healthy aging [19]. Moreover, Johnson et al. (2015) have demonstrated that EPA but not DHA, attenuated the age-related loss of mitochondrial function in skeletal muscle of old mice [16]. It has also been shown that DHA and EPA influenced expression of anti-oxidant enzymes of mouse skeletal muscle cells [20] and decreased mitochondrial reactive oxygen species (ROS) production in skeletal muscle of older adults after four months of a n-3 PUFA (mix of EPA and DHA) dietary intervention [21]. Therefore, mitochondrial metabolism seems to be a prime target for the beneficial effects of n-3 PUFAs during aging. However, aging is a progressive process and contradictory results exist in the literature about the effects of either EPA or DHA on mitochondrial functions [15,16,20,21]. We hypothesized that these discrepancies can partly be explained by (i) the fact that EPA and DHA can be interconverted, which makes difficult to pinpoint their specific effects, (ii) the model used, as the experimental time-frame to evaluate the effects of n-3 PUFA in the context of aging can be problematic in rodents or humans.

The aim of the current study was to determine the effects of dietary n-3 PUFA supplements on the mitochondrial metabolism and oxidative stress of *Drosophila melanogaster* sampled at five different ages. Two different supplements were specifically tested: (i) a 40.08% monoacylglyceride rich oil enriched in DHA (65% *w*/*w*), referred as MAG-DHA; (ii) a 40.72% monoacylglyceride rich oil enriched in EPA (82% *w*/*w*), referred as MAG-EPA. These monoacylglyceride rich oils comply with the USP monograph of mono- and di-acylglycerides (see Certificates of analysis in supplementary material). Although these compounds are a heterogenous mixture of mono- and diacylglycerides, they were chosen in our study based on their high contents in monoacyglycerides (more than 40%). These compounds have been shown to increase the plasma concentration and bioavailability of both EPA and DHA n-3 PUFAs in rodents compared to other forms [22] and have never been tested in Drosophila. This short-lifespan model has recently emerged as a suitable model to understand the fundamental mechanisms that control metabolism [23,24,25,26,27] and does not possess the Δ^5^ and Δ^6^ desaturases which participate in the conversion of ALA to EPA and of EPA to DHA [28,29]. Here, we show that although both MAG-DHA and MAG-EPA significantly increased longevity in Drosophila, MAG-EPA has more potent effects on mitochondrial respiration and provides protection against lipid peroxidation by maintaining superoxide dismutase activity during aging. We suggest that investigating the metabolic pathways of DHA and EPA in Drosophila can provide new understanding about the potential beneficial effects of these essential n-3 PUFAs on several pathological conditions occurring during aging.

## 2. Results

### 2.1. MAG-DHA and MAG-EPA Extend Longevity in D. melanogaster

**Figure 1:**
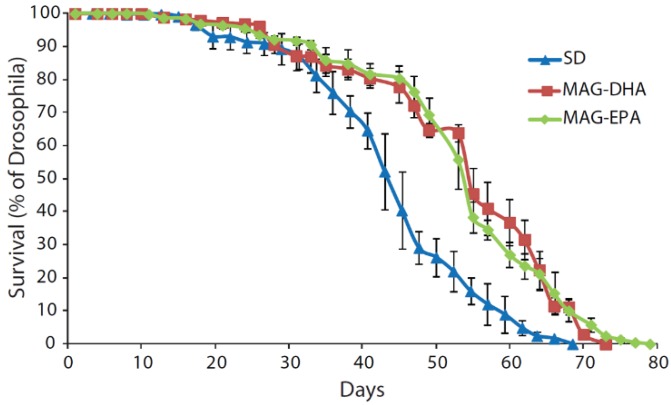
Survival curve of *Drosophila melanogaster* males fed a standard diet (SD, blue), a standard diet supplemented with MAG-DHA (red) and a standard diet supplemented with MAG-EPA (green). Results are presented as the percentage of Drosophila alive counted every 2–3 days (*N* > 145 for each group).

Male Drosophila (strain w^1118^, Bloomington Drosophila Stock Center, Bloomington, IN, USA) were collected on the day of hatching and were fed a standard cornmeal diet (SD), or a SD supplemented with 0.3 mg·mL^−1^ of either MAG-DHA or MAG-EPA. This concentration was determined in accordance with another study showing effects of a DHA-rich marine microalga on Drosophila longevity [30]. The longevity is presented in Figure 1 and was evaluated by recording the survival of flies every 2–3 days (*N* > 145, in triplicates). The three groups were significantly different from each other (log-rank χ^2^ = 16.5, *P* < 0.001 between SD and MAG-DHA; log-rank χ^2^ = 48.3, *P* < 0.001 between SD and MAG-EPA; log-rank χ^2^ = 9.8, *P* = 0.002 between MAG-DHA and MAG-EPA). Specifically, median lifespans were similar between MAG-DHA and MAG-EPA (55 days) and both were higher than when the flies were fed the SD (48 days). Maximal lifespan was however the highest with MAG-EPA (79 days), followed by MAG-DHA (73 days) and SD (68.5 days).

### 2.2. MAG-DHA and MAG-EPA Decrease Mitochondrial Proton Leak and Ameliorate Mitochondrial Coupling

Mitochondrial oxygen consumption was evaluated in permeabilized thorax of Drosophila at five different ages (15, 25, 30, 35 and 45 days old, *N* = 5–6 for each dietary treatment at each age). We only started to measure mitochondrial respiration at 15 days-old because it usually takes 10 to 15 days for all larval fat cells to be removed in Drosophila [31], which could have biased the results. All respiration rates determined using substrates to stimulate different components of the electron transport system was affected by the dietary treatment, the age and/or the interaction treatment * age (Table 1).

**Figure 2:**
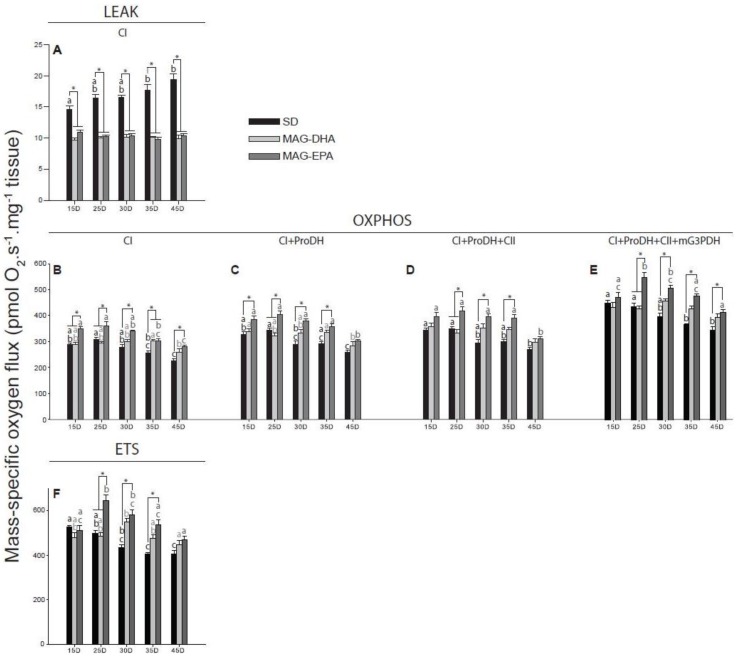
Effects of n-3 PUFAs on mass-specific mitochondrial respiration rates of thorax muscle from *Drosophila melanogaster*. Mitochondrial respiration rates were measured during (**A**) the LEAK respiration in presence of pyruvate+malate (CI-LEAK); (**B**–**E**) the OXPHOS respiration after addition of ADP (CI-OXPHOS), proline (CI+ProDH-OXPHOS), succinate (CI+ProDH+CII-OXPHOS) and glycerol-3-phosphate (CI+ProDH+CII+mG3PDH-OXPHOS); (**F**) and the non-coupled respiration after injection of FCCP ETS). Respiration rates were measured in flies fed a standard diet (SD, black bars), a standard diet supplemented with MAG-DHA (light grey) and a standard diet supplemented with MAG-EPA (dark grey) at 15, 25, 30, 35 and 45 days old (*N* = 5–6 for each dietary treatment at each age). Results are means ± s.e.m. Dissimilar letters represent significant differences between ages of the same dietary treatment. * denotes significant differences between dietary treatments at the same age. Significance was set at *P* < 0.05.

First, pyruvate and malate were used to monitor the leak respiration at the level of complex I (CI-LEAK), which corresponds to the mitochondrial oxygen consumption compensating for the proton leak through the inner mitochondrial membrane without ADP phosphorylation. CI-LEAK of Drosophila fed either MAG-DHA or MAG-EPA were lower than with the SD across all the ages tested (for all ages, all *P*-values < 0.001 for comparisons between MAGs and SD; Figure 2A). Moreover, while CI-LEAK was similar across all ages for both MAG-DHA and MAG-EPA, it was increased for SD at 35 and 45 days old when compared to 15 days old (*P* = 0.016 and *P* < 0.001, respectively; Figure 2A). The addition of ADP allowed to measure the mitochondrial oxygen consumption at the level of complex I during phosphorylation of ADP into ATP (CI-OXPHOS, Figure 2B). For all the groups tested, the same trend was observed, with a decrease of CI-OXPHOS occurring at 45 days old (SD: *P* < 0.001 between 15–25 and 45 days and *P* = 0.004 between 30 and 45 days; MAG-DHA: *P* = 0.041 between 35 and 45 days; MAG-EPA: *P* < 0.001 between 15-25 and 45 days and *P* = 0.001 between 30 and 45 days). Moreover, flies fed MAG-EPA presented higher CI-OXPHOS than SD for all ages tested (*P* < 0.001, *P* = 0.002, *P* < 0.001, *P* = 0.030 and *P* = 0.006 at 15, 25, 30, 35 and 45 days old, respectively) and MAG-DHA at 15 and 25 days old (all *P*-values < 0.001), while MAG-DHA was also higher than SD at 35 days old (*P* = 0.036).

**Figure 3:**
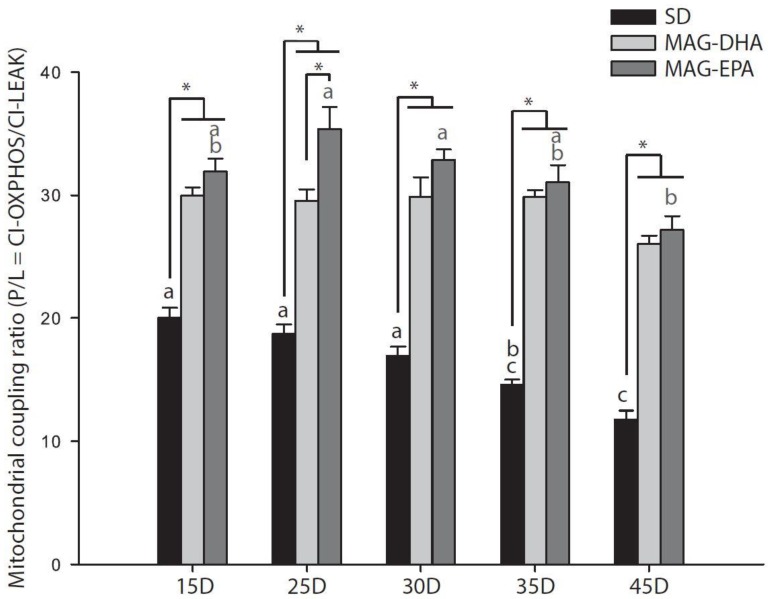
Effects of n-3 PUFAs on the mitochondrial coupling ratio calculated from mass-specific respiration rates measured in permeabilized thoraxes from *Drosophila melanogaster* males. Mitochondrial coupling ratio at the level of complex I (P/L) was calculated as CI-OXPHOS/CI-LEAK in flies fed a standard diet (SD, black bars), a standard diet supplemented with MAG-DHA (light grey) and a standard diet supplemented with MAG-EPA (dark grey) at 15, 25, 30, 35 and 45 days old (*N* = 5–6 for each dietary treatment at each age). Results are means ± s.e.m. Dissimilar letters represent significant differences between ages of the same dietary treatment. * denotes significant differences between dietary treatments at the same age. Significance was set at *P* < 0.05.

Interestingly, the coupling ratio (P/L = CI-OXPHOS/CI-LEAK; Table 1, Figure 3), which is an indicator of the coupling between electron transport and phosphorylation of ADP [32] was significantly decreased for SD at 35 compared to 15 days old (*P* = 0.017) and was further decreased at 45 days old (all *P*-values < 0.001 when compared to 15, 25 and 30 days old).

On the other hand, no decreases were detected in the P/L ratio for MAG-DHA (Figure 3) and a significant decrease was detected for MAG-EPA between 25–30 and 45 days old (*P* < 0.001 and *P* = 0.019 for 25 and 30 days old respectively) due to a small increase of the P/L ratio at 25 and 30 days old (Figure 3). Both MAG-DHA and MAG-EPA also had higher P/L ratio than SD at all ages tested (all *P*-values < 0.001) and MAG-EPA presented higher P/L ratio than MAG-DHA at 25 days old (*P* = 0.008).

### 2.3. MAG-EPA Generally Increases Mitochondrial Oxidative Capacities

Other contributors of the electron transport system that allows the stimulation of mitochondrial oxygen consumption were then evaluated by sequentially injecting several substrates [33]: proline dehydrogenase (ProDH), succinate dehydrogenase (complex II) and mitochondrial glycerol-3-phosphate dehydrogenase (mtG3PDH) which provide electrons from proline, succinate and glycerol-3-phosphate, respectively (respiration rates: CI+ProDH-OXPHOS, CI+ProDH+CII-OXPHOS and CI+ProDH+CII+mtG3PDH-OXPHOS, Figure 2C–E). For the SD a progressive decline of the different respiration rates measured was observed with aging (Figure 2C–E). Specifically, CI+ProDH-OXPHOS was significantly decreased at 45 days old (*P* < 0.001 when compared to either 15 or 25 days old), as well as between 25 and 30 days old (*P* = 0.038); CI+ProDH+CII-OXPHOS was decreased at 45 days old (*P* =0.004 and *P* = 0.001 when compared to 15 and 25 days old, respectively); and CI+ProDh+CII+mG3PDH-OXPHOS was decreased at 35 days old (*P* < 0.001 and *P* = 0.015 when compared to 15 and 25 days old, respectively), as well as at 45 days old (*P* < 0.001 when compared to either 15 or 25 days old).

For flies fed MAG-DHA a decrease was also observed for CI+ProDH-OXPHOS at 45 days old (*P* = 0.031 and *P* = 0.046 when compared to 15 and 35 days old, respectively; Figure 2C) but no significant declines were detected for CI+ProDH+CII-OXPHOS and CI+ProDH+CII+mG3PDH-OXPHOS (Figure 2D,E). When fed MAG-EPA, the 45 days old flies also displayed significant decreases of CI+ProDH-OXPHOS (*P* < 0.001 for comparisons with 15, 25 and 30 days old; *P* = 0.029 with 35 days old; Figure 2C) and CI+ProDH+CII-OXPHOS (*P* < 0.001 for comparisons with 15, 25 and 30 days old; *P* = 0.004 with 35 days old; Figure 2D). However, at the level of CI+ProDH+CII+mG3PDh-OXPHOS (Figure 2E) an increase was first detected between 15 and 25 days old (*P* = 0.003), followed by a progressive decline from 25 to 45 days old with a significant decrease observed between 25 and 35 days old (*P* = 0.009), between 25 and 45 days old (*P* < 0.001), as well as between 30 and 45 days old (*P* < 0.001). The decreased values for CI+ProDH+CII+mG3PDH were however similar to those obtained at 15 days old (Figure 2E).

When comparing the different diets, flies fed MAG-EPA had higher oxidative capacities than those fed the SD for CI+ProDH-OXPHOS at 15, 25, 30 and 35 days old (*P* = 0.018, *P* = 0.005, *P* < 0.001 and *P* = 0.002, respectively), for CI+ProDh+CII-OXPHOS at 25, 30 and 35 days old (*P* = 0.009, *P* < 0.001 and *P* < 0.001, respectively), as well as for CI+ProDh+CII+mG3PDH-OXPHOS at 25, 30, 35 and 45 days old (*P* < 0.001, *P* < 0.001, *P* < 0.001 and *P* = 0.030, respectively). Moreover, significant increases of these respiration rates were also detected with MAG-EPA compared to MAG-DHA but only at 25 days old (all *P*-values < 0.001).

### 2.4. Flies Fed MAG-EPA Have Higher Electron Transport System Capacity

The protonophore carbonyl cyanide-*4*-(trifluoromethoxy)phenylhydrazone (FCCP) was then injected stepwise, enabling the transport of protons from the intermembrane space to the mitochondrial matrix without passing through complex V. This respiration rate represents the non-coupled respiration that is, the maximal capacity of the electron transport system (ETS, Figure 2F). With the SD, a significant decline of ETS capacity was observed at 30 days old (15 vs. 30 days old, *P* = 0.030), 35 days old (*P* < 0.001 and *P* = 0.027 when compare to 15 and 25 days old, respectively) and 45 days old (*P* < 0.001 and *P* = 0.027 when compare to 15 and 25 days old, respectively; Figure 2F). Flies fed MAG-DHA did not display this decline and ETS capacity was augmented at 30 days old (only significantly with 45 days old, *P* = 0.015; Figure 2F). For MAG-EPA, a small but not significant decrease was observed between 15 and 45 days old and ETS capacity was increased at 25 days old (*P* < 0.001, *P* = 0.005 and *P* < 0.001 when compared to 15, 35 and 45 days old, respectively) and at 30 days old (*P* = 0.005 when compared to 45 days old; Figure 2F). Moreover, flies fed MAG-EPA had higher ETS capacity than SD at 25, 30 and 35 days old (all *P*-values < 0.001), as well as when compared to MAG-DHA at 25 days old (*P* < 0.001; Figure 2F).

### 2.5. MAG-EPA Delays the Onset of Oxidative Stress

**Figure 4:**
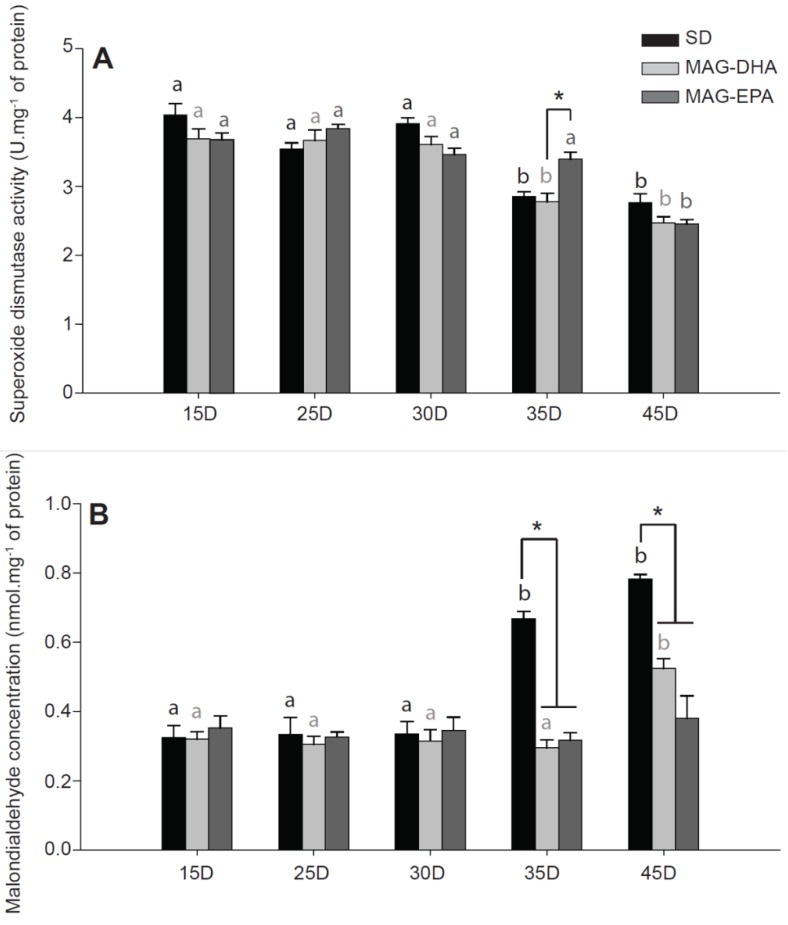
Markers of oxidative stress in thorax muscle of *Drosophila melanogaster* after a n-3 PUFAs dietary intervention. (**A**) Superoxide dismutase activity and (**B**) Malondialdehyde (MDA) concentration measured in flies fed a standard diet (SD, black bars), a standard diet supplemented with MAG-DHA (light grey) and a standard diet supplemented with MAG-EPA (dark grey) at 15, 25, 30, 35 and 45 days old (*N* = 6 for each dietary treatment at each age). Results are means ± s.e.m. Dissimilar letters represent significant differences between ages of the same dietary treatment. * denotes significant differences between dietary treatments at the same age. Significance was set at *P* < 0.05.

It is now well-known that the level of oxidative stress tends to increase during aging [34]. To determine oxidative stress during aging in our dietary treatments, we measured in homogenates from thorax of Drosophila the total activity of the antioxidant enzyme superoxide dismutase (SOD), as well as the concentration of malondialdehyde (MDA) which is one of the end-products of lipid peroxidation (*N* = 6 for each dietary treatments at each age; Figure 4).

In both SD and MAG-DHA flies, total SOD activity was significantly decreased at 35 (all *P*-values < 0.001 for 15, 25 and 30 days old for both dietary treatments) and at 45 days old (all *P*-values < 0.001 for 15, 25 and 30 days old for both dietary treatments; Figure 4A). In MAG-EPA flies, a significant decrease was also detected but only at 45 days old (all *P*-values < 0.001 for 15, 25, 30 and 35 days old). Moreover, the MAG-EPA flies had higher total SOD activity at 35 days old compared to MAG-DHA flies (*P* = 0.002; Figure 4A).

Oxidative damage to lipids, as estimated by MDA levels, were drastically increased in SD flies at 35 and 45 days old (all *P*-values < 0.001 when both ages were compared to 15, 25 and 30 days old; Figure 4B). In flies fed MAG-DHA a significant increase was also observed at 45 days old (*P* = 0.003, *P* = 0.001, *P* = 0.002 and *P* < 0.001 for comparisons with 15, 25, 30 and 35 days old, respectively; Figure 4B). However, no significant differences were detected in MDA levels for flies fed MAG-EPA during aging (Figure 4B). The drastic increase of MDA levels in SD flies at both 35 and 45 days old was also significant when compared to either MAG-DHA flies or MAG-EPA flies (all *P*-values < 0.001).

## 3. Discussion

In this study, we show that supplementation of dietary n-3 PUFA monoacylglycerides caused an increased lifespan of Drosophila males and modulated mitochondrial oxidative capacity and markers of oxidative stress in thorax muscle. Specifically, both MAG-DHA and MAG-EPA cause a decrease of mitochondrial proton leak resulting in an increase of mitochondrial coupling but only MAG-EPA improved the ETS capacity and had more potent effects on mitochondrial oxidative capacities. Moreover, both n-3 PUFAs delayed the occurrence of lipid peroxidation. However, MAG-EPA had greater protective effects against oxidative damages likely due to a better preservation of total SOD activity during aging. Therefore, our study provides evidence that the mitochondrial metabolism of Drosophila is generally improved with MAG-EPA, leading to the delay of senescence which is reflected by an increased longevity. Moreover, our results suggest that Drosophila could be a relevant model for the study of the fundamental mechanisms linking the effects of n-3 PUFAs to aging.

In Drosophila, it has been shown that a DHA-rich marine microalgae (around 0.53 mg·mL^−1^ of DHA in the diet) caused a 10% increase of median and a 11% increase of maximum lifespan [30]. In our study, MAG-DHA increased by 14.6% and 6.6% median and maximum lifespan, respectively, while MAG-EPA increased median and maximum lifespan by 14.6% and 15.3%, respectively (Figure 1). Interestingly, it has been demonstrated that Drosophila naturally lacks DHA and EPA and that DHA supplementation caused an important increase of EPA, suggesting a 85% retroconversion of DHA to EPA [28]. Indeed, while Δ^5^ and Δ^6^ desaturases required for the conversion of ALA to EPA and of EPA to DHA seems to be absent in Drosophila [28], they possess enzymes allowing the peroxisomal oxidation of DHA to EPA [35]. It is therefore possible that the majority of the longevity effects seen with DHA were mediated by its oxidation to EPA.

Both MAG-DHA and MAG-EPA drastically decreased the mitochondrial oxygen consumption compensating for the proton leak (CI-LEAK; Figure 2A). The fatty acid composition of mitochondrial membranes is an important determinant of proton leak, as membranes with higher PUFA levels are associated with higher rates of proton leak [36]. Moreover, incorporation of DHA and EPA into mitochondrial membranes has been demonstrated after supplementation in muscles of healthy men [14]. Thus, incorporation of DHA and EPA to mitochondrial membranes would theoretically cause an increase of proton leak, which is not consistent with our results. However, it has recently been shown that a supplementation of n-3 PUFA (mix of DHA and EPA) reduced proton leak in muscles of old men and women, although with different substrates we used in our study [21]. It has been suggested that proton leak may serve to decrease ROS production, especially in ectotherms [37]. A possible explanation for our results would therefore be that flies fed with the SD have higher endogenous ROS production than those fed MAG-DHA or MAG-EPA even at younger ages and have to increase their CI-LEAK to minimize oxidative damages. While we did not specifically measure ROS production, other studies have shown that H_2_O_2_ production was reduced by 20–25% after 4 months of n-3 PUFA consumption in muscles of older adults when the ROS-emitting potential of mitochondria was the highest, that is, low CI-LEAK [21]. Moreover, CI-LEAK was also augmented with the SD during aging (Figure 2A). Although some studies have reported increased proton leak during aging [38], others showed a general decrease of proton leak with aging [16,21,39]. However, it has been shown in Drosophila that proton leak is either stable or slightly augmented with aging [40,41,42] and it is therefore possible that our results reflect the propensity of Drosophila to increase their proton leak during aging to alleviate the deleterious effects of increased ROS production.

When the OXPHOS state was determined by allowing the phosphorylation of exogenous ADP to ATP (Figure 2B–E), the same trend was observed for all the dietary treatments, with the mitochondrial oxidative capacities of different substrates generally decreasing at 45 days old. However, this decline was more pronounced in flies fed the SD and was apparent at younger ages (Figure 2B–E). Moreover, while MAG-DHA does not increase mitochondrial oxidative capacities, flies fed MAG-EPA displayed higher mitochondrial oxygen consumption at almost all ages compared to those fed the SD. Notably, at 45 days old, CI-OXPHOS and CI+ProDH+CII+mG3PDH-OXPHOS were increased with MAG-EPA. The same trend was observed for the non-coupled respiration (ETS, Figure 2F), suggesting that the overall capacity to transfer electrons from one complex to another inside the inner mitochondrial membrane is higher when flies are fed MAG-EPA. In a recent study, it has been demonstrated that EPA but not DHA, restores muscle mitochondrial oxidative capacities of old mice [16], which is in accordance with our results. However, Herbst et al. (2014) showed that in healthy young men, fish oil supplementation (mix of EPA and DHA) did not change mitochondrial respiratory functions but improved mitochondrial ADP kinetics [14], which contrasts with our study as we observed a general increase in mitochondrial oxidative capacities even at younger ages. A possible explanation for our results would be that the differences observed in mitochondrial capacities with MAG-EPA are exacerbated in Drosophila compared to humans because carbohydrate-derived substrates (such as pyruvate and glycerol-3-phosphate) are preferentially used in Drosophila. An alternative explanation would be that monoacylglycerides have more potent effects on mitochondrial respiration than other forms of n-3 PUFAs. Indeed, oral supplementation of n-3 PUFA monoacylglycerides have been shown to increase the plasma concentration and bioavailability of these n-3 PUFAs in rodents compared to other forms [22] but this hypothesis has to be properly tested before being validated. Interestingly, a study on Drosophila demonstrated that after chill coma, higher mitochondrial capacities and improved climbing abilities with arachidonic acid, as well as shorter recovery time with docosahexaenoic acid were detected [43], which seems to be consistent with our study. Moreover, lifespan was decreased with arachidonic acid, suggesting that polyunsaturated fatty acids can be detrimental to Drosophila [43]. However, only arachidonic acid and ALA were detected in the flies after exposure to the diet with arachidonic acid [43]. It therefore suggests that while ALA or arachidonic acid cannot participate to increased lifespan in Drosophila, the lifespan effect detected in our study should be specific to EPA and/or DHA. A future study comparing the effect of EPA, DHA, ALA and arachidonic acid on Drosophila lifespan could confirm this assumption.

The combination of a decreased proton leak with n-3 PUFAs and an increased CI-OXPHOS with MAG-EPA led to significantly higher mitochondrial coupling than SD for both MAG-DHA and MAG-EPA (Figure 3). It is well-known that aging tends to decrease this coupling ratio (often referred to mitochondrial coupling efficiency or respiratory control ratio) and while the expected decrease was observed with the SD, it was less apparent with either MAG-DHA or MAG-EPA. Johnson et al. (2015) showed that the coupling ratio was also restored in old mice supplemented with EPA but not with DHA [16]. Our results showed that both n-3 PUFAs increased the coupling ratio at all ages and maintain this coupling during aging. Altogether, the results for mitochondrial oxidative and coupling capacities indicate that mitochondrial functions are improved when flies are fed MAG-EPA. However, it is possible that this improvement reflects a quantitative (more mitochondria) rather than a qualitative adjustment. Indeed, it has been suggested that n-3 PUFAs stimulate mitochondrial biogenesis through activation of transcription factors [44,45,46]. We therefore measured citrate synthase activity and oxygen consumption of complex IV (with TMPD and ascorbate), which have been shown to be good markers of mitochondrial content [47,48]. We did not find that these markers were affected by the interaction dietary treatment*age and were not increased in flies fed n-3 PUFAs (Table 1; Figure S1), suggesting that the differences observed in mitochondrial oxidative and coupling capacities were due to modulation of mitochondrial functions per se, consistent with other studies [14,16,46].

Interestingly, n-3 PUFAs have often been associated to antioxidant effects in different experimental models (reviewed in Reference [49]). Specifically, it has been shown that diet containing EPA and DHA in different ratios may increase the expression of anti-oxidant enzymes and notably of SOD [30,50] and decrease level of oxidative damages [45,49,51]. Our results showed that total SOD activity was decreased at 35 days old with SD and MAG-DHA but was only decreased at 45 days old for flies fed MAG-EPA (Figure 4A). Moreover, lipid peroxidation increased in SD flies at 35 days old and in MAG-DHA flies at 45 days old but not in MAG-EPA flies, as indicated by MDA levels (Figure 4B). These results suggest that MAG-EPA and to a lesser extend MAG-DHA, had protective effects against oxidative damages during aging but do not affect anti-oxidant capacities at younger ages. This protective effect on mitochondrial functions and redox status is in accordance with another study showing that in Drosophila exposed to paraquat (a well-known inducer of oxidative stress), EPA/DHA supplements restored mitochondrial functions and inhibit H_2_O_2_ production [52].

In conclusion, our results demonstrate that n-3PUFAs modulate mitochondrial functions and anti-oxidant capacities of Drosophila thorax muscle during aging. Notably, MAG-EPA have more potent effects than MAG-DHA, which translates into increased mitochondrial oxidative capacities and better protection against oxidative damages in old flies. In turn, these improved capacities could explain the increased lifespan observed in Drosophila. Minor effects were also detected with MAG-DHA, as well as a similar increased longevity than with MAG-EPA. Considering the important retroconversion of DHA to EPA in Drosophila [28], it is therefore likely that these effects were the results of a major oxidation of DHA to EPA. Future studies using uniformly labeled carbon-13 DHA and EPA [53] in Drosophila would allow the investigation of this metabolic conversion and could provide important information on n-3 PUFA metabolism in this animal model. Although we cannot ascertain the precise effects of MAG-EPA, our study suggests that mitochondrial metabolism is primarily modulated by this n-3 PUFA. These effects could be related to changes in mitochondrial membrane composition or to post-translational modifications of mitochondrial enzymatic complexes and/or of anti-oxidant enzymes, as already suggested [14,16,21]. Another possibility would be that n-3 PUFAs affect the regulation of mitochondrial ROS. Since n-3 PUFA decreased proton leak (which is involved in the modulation of ROS production [37]) and had an effect on SOD activity, one interesting research avenue would be to evaluate the contribution of n-3 PUFA on mitochondrial ROS production/detoxification during aging in Drosophila. Additionally, we demonstrated that Drosophila could be a relevant model for the metabolism of n-3 PUFAs, as physiological and metabolic effects can be detected in this organism after a dietary intervention. This is particularly interesting considering that EPA cannot be converted to DHA in flies but the retroconversion of DHA to EPA can still occur. Therefore, the biological effects of each individual n-3 PUFA and particularly of EPA, can be determine in the context of aging using Drosophila.

## 4. Materials and Methods

### 4.1. Synthesis of n-3 PUFA Monoacylglycerides

*MAG-DHA and MAG-EPA*. MAG-DHA and MAG-EPA were obtained by hemisynthesis from a blend of anchovy, sardine and mackerel body oil. Briefly, the blended oil was concentrated to obtain a docosahexaenoic acid rich oil (65% *w*/*w*) or an eicosatetraenoic acid rich oil (82% *w*/*w*). The resulting oils were reacted with glycerol and a lipase to obtain a monoglycerides rich oil (MAG-DHA or MAG-EPA) that complies with the USP monograph of Mono- and Di-glycerides. Certificates of analysis can be found as supplementary material [54].

### 4.2. Drosophila Model and Longevity

*Drosophila melanogaster* w^1118^ (Bloomington Drosophila Stock Center, Bloomington, IN, USA) were maintained at constant temperature (24.0 ± 0.1 °C), humidity (50% relative humidity) and diurnal cycle (12:12 h light:dark) and were fed on a standard cornmeal medium (SD: 5 g agar-agar, 6 g sugar, 27.5 g dried yeast and 53 g cornmeal flour dissolved in 1 L of tap water, with 4 mL propionic acid, 16 mL methyl P-hydroxybenzoate [10% *w*/*v*] added to the mixture to avoid mite and mold contamination). Males were collected the day of hatching and were transferred at constant densities to SD, or to SD supplemented with 0.3 mg·mL^−1^ of either MAG-DHA or MAG-EPA. This concentration was chosen according to Huangfu et al. [30]. For longevity experiments, the number of flies alive was recorded after the transfer to the dietary treatments every 2–3 days (*N* > 145) and the experiments were repeated three times. Flies were transferred to fresh food every 5–7 days. For the other experiments, flies were sampled at the days of interest (15, 25, 30, 35 and 45 days old) and were either directly processed for measurement of mitochondrial oxygen consumption or frozen in liquid nitrogen and kept at −80 °C for further biochemical assays.

### 4.3. Thorax Permeabilization and Mitochondrial Respiration

Permeabilization of thorax and measurement of mitochondrial oxygen consumption at 24 °C (*N* = 6 for each day and for each treatment) were performed as previously described [55,56,57]. Briefly, thoraxes were dissected and were permeabilized mechanically and chemically (using saponin) and were transferred to an Oxygraph-O2K (Oroboros Instruments, Innsbruck, Austria). Mitochondrial oxygen consumption was measured after sequential injections of different substrates: 5 mM pyruvate +2 mM malate (CI-LEAK); +5 mM ADP (CI-OXPHOS); +15 μM cytochrome c (to verify to integrity of the outer mitochondrial membrane); +5 mM proline (CI+ProDH-OXPHOS); +20 mM succinate (CI+ProDH+CII-OXPHOS); +15 mM glycerol-3-phosphate (CI+ProDH+CII+mG3PDH-OXPHOS); +0.5–1 μM steps of FCCP (CI+ProDH+CII+mG3PDH-ETS). Subsequent inhibitions of complexes I, II and III by rotenone (0.5 μM), malonate (5 mM) and antimycin A (2.5 μM) were performed to evaluate the residual oxygen consumption which was used to correct the previous respiration rates measured.

### 4.4. Oxidative Stress Markers

Drosophila were homogenized in a 50 mM MES, 1 mM EDTA, pH 7.2 buffer and were centrifuged at 1500× *g* for 7 min at 4 °C. The resulting supernatant was assayed for total SOD activity and MDA levels that were normalized to total protein content measured with the bicinchoninic acid method.

#### 4.4.1. Superoxide Dismutase Activity

Total SOD activity was measured at 24 °C using a Superoxide Dismutase Assay kit from Cayman Chemical (Ann Harbor, MI, United States) following the manufacturer protocol. Briefly, this assay follows the superoxide radicals generated by xanthine oxidase and hypoxanthine using tetrazolium salt for spectrophotometric detection at 450 nm. Total SOD is expressed as means of U·mg^−1^ proteins ± s.e.m. where one unit of SOD is defined as the amount of enzyme needed to exhibit 50% dismutation of the superoxide radical.

#### 4.4.2. MDA Levels

MDA levels were measured using the TBARS assay kit from Cayman Chemical (Ann Harbor, MI, USA). Briefly, the samples were incubated with thiobarbituric acid at high temperature (90–100 °C) and the adducts formed by the reaction were determined fluorimetrically at an excitation wavelength of 530 nm and an emission wavelength of 550 nm against a standard curve of MDA. Results are expressed as nmol of MDA formed per mg of proteins ± s.e.m.

### 4.5. Statistical Analyses

All statistical analyses were performed with R software (version 3.1.0, Free Software Foundation, Boston, MA, USA). For longevity, a log-rank test was performed to detect survival differences between the different dietary treatments. For mass-specific mitochondrial respiration rates, P/L ratios, total SOD activity and MDA levels, the data were fitted to a linear model and were analyzed using a two-way ANOVA (type 2) with the treatment and the age as fixed factors. Multiple comparisons were then tested with pairwise comparisons of the least-squares means using adjusted *P*-values (Tukey method) with significance set at *P* < 0.05. Normality was verified with the Shapiro-Wilk’s test and homogeneity of variances was verified using the Levene’s test and data were transformed when required.

## Figures and Tables

**Table 1:** Analyses of variance for *Drosophila melanogaster* males exposed to different diets (SD, supplemented with MAG-DHA and supplemented with MAG-EPA) and aged 15, 25, 30, 35 and 45 days old.

	Denominator *df*	Dietary Treatment *df* = 2	Age *df* = 4	Dietary Treatment * Age *df* = 8
**Respiration Rates**				
CI-LEAK	73	30.68 ***	8.57 ***	4.00 ***
CI-OXPHOS	73	16.58 ***	12.88 ***	2.88 **
CI+ProDH-OXPHOS	73	8.56 ***	10.52 ***	2.51 *
CI+ProDH+CII-OXPHOS	73	5.47 **	7.88 ***	2.31 *
CI+ProDH+CII+mG3PDH-OXPHOS	73	2.24	12.52 ***	4.67 ***
ETS	73	1.90	9.83 ***	6.53 ***
Complex IV	73	0.009	11.94 ***	0.55
**P/L for Complex I**	73	40.71 ***	10.98 ***	2.11 *
**Citrate Synthase Activity**	75	13.65 ***	8.78 ***	0.64
**Oxidative Stress Markers**				
Superoxide dismutase activity	75	4.34 *	26.03 ***	4.57 ***
Malondialdehyde concentration	75	0.29	44.07 ***	12.11 **

* *P* < 0.05; ** *P* < 0.01; *** *P* < 0.001.

## Supplementary Materials

The following are available online at http://www.mdpi.com/1660-3397/16/11/453/s1. Supplementary material includes supplementary methods (Integrity of mitochondrial outer membrane; Complex IV capacity; and Citrate synthase activity), one supplementary figure (Figure S1: Effects of n-3 PUFAs on markers of mitochondrial content in thorax muscle from Drosophila melanogaster), the certificate of analysis for MAG synthesis and the datasets used in this manuscript.
