# Supplementary material for: Omega-3 Monoacylglyceride Effects on Longevity, Mitochondrial Metabolism and Oxidative Stress: Insights from Drosophila melanogaster
Source: Mar Drugs. 2018 Nov 16;16(11):453. doi: 10.3390/md16110453 (PMC6266923; doi:10.3390/md16110453)
Supplement: Supplementary file 1 [file marinedrugs-16-00453-s001.pdf]

marine drugs

Supplementary Material

Supplementary Methods

Integrity of Mitochondrial Outer Membrane

After ADP was injected into the chambers of the respirometer (CI-OXPHOS, see experimental procedures), 15 µM cytochrome c were added to verify the integrity of the outer mitochondrial membrane and was used as a quality control of the mitochondrial preparations. Cytochrome c is loosely bound to the inner mitochondrial membrane and is typically washed away if the outer mitochondrial membrane is damaged during the permeabilization process. As a result, adding exogenous cytochrome c will significantly increase oxygen consumption if the outer mitochondrial membrane is damaged and the endogenous cytochrome c is lost. An increase of less than 10–15% in oxygen consumption usually illustrates appropriate integrity of the outer mitochondrial membrane [1]. An increase of less than 5 % was observed for all the samples used in our study, attesting good permeabilization and integrity of the mitochondrial outer membrane.

Complex IV Capacity

After sequential inhibition of mitochondrial complexes I, II and III (see experimental procedures), ascorbate (2 mM) and N,N,N′,N,-Tetramethyl-p-phenylenediamine (TMPD, 0.5 mM) were added to evaluate the maximum capacity of complex IV (CIV). TMPD is an artificial electron transporter that provides electrons directly to complex IV, but is prone to autoxidation. To correct for this autoxidation, Complex IV was inhibited with 20 mM of sodium azide and the remaining oxygen consumption was taken into account for the calculation of complex IV maximal respiration capacity. This respiration rate was used as a proxy for mitochondrial content [2].

Citrate Synthase Activity

Citrate synthase (CS) activity was determined in thorax homogenates of flies (N = 6 for each dietary treatments at each age) as a marker of mitochondrial content [2]. CS activity was measured with a Varioskan™ microplate reader (ThermoScientific™) at 412 nm for 4 min by following the reduction of 5,5′-dithiobis-2-nitrobenzoic acid (DTNB, ε = 13.6 $mL \cdot cm^{-1} \cdot umol^{-1}$) using a 100 mM imidazole-HCl buffer containing 0.1 mM DTNB, 0.1 mM AcetylCoA and 0.15 mM oxaloacetic acid (omitted from the blank), pH 8.0. CS activity is expressed as $U \cdot mg^{-1}$ protein, where U represents 1 µmol of substrate transformed to product in 1 min.

Supplementary Figures

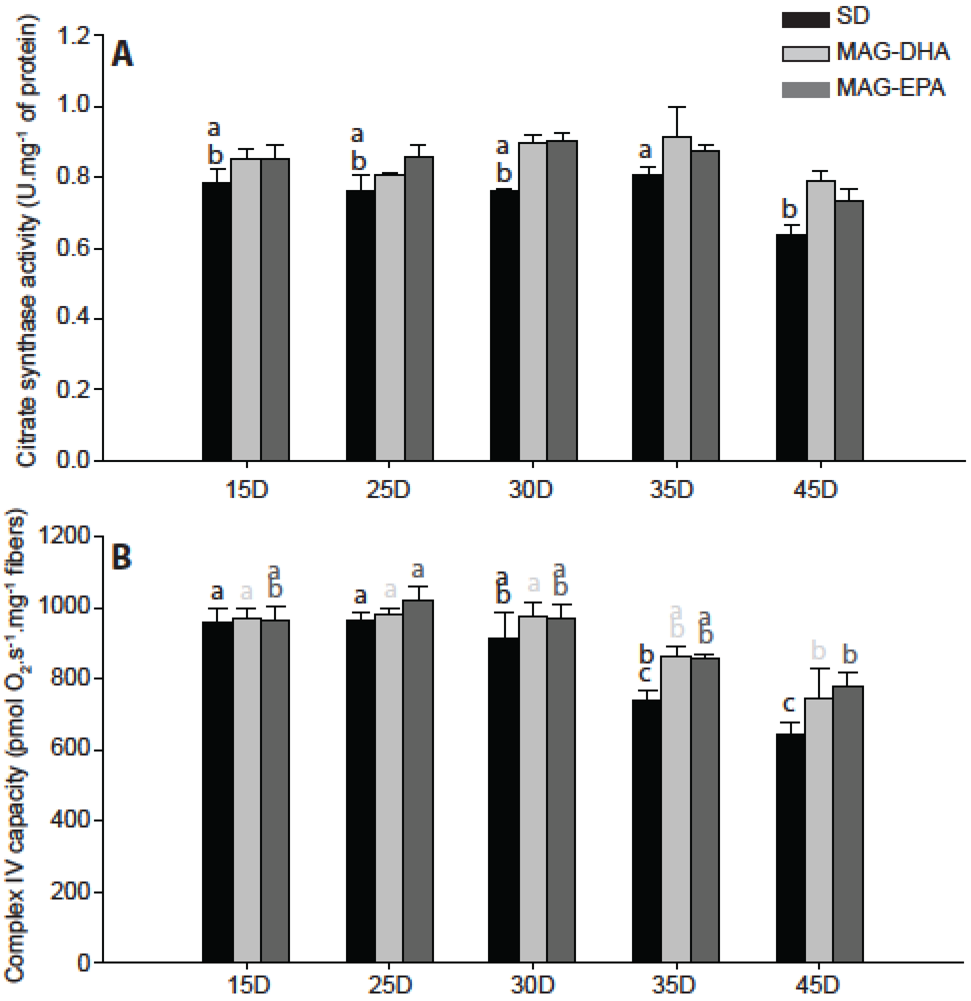

Figure S1. Effects of n-3 PUFAs on markers of mitochondrial content in thorax muscle from *Drosophila melanogaster*. (**A**) Citrate synthase (CS) activity and (**B**) Complex IV maximal capacity. Both parameters were measured in flies fed a standard diet (SD, black bars), a standard diet supplemented with MAG-DHA (light grey), and a standard diet supplemented with MAG-EPA (dark grey) at 15, 25, 30, 35, and 45 days old (N = 6 and N = 5–6 for CS activity and complex IV maximl capacity, respectively, for each dietary treatment at each age). Results are means ± s.e.m. Dissimilar letters represent significant differences between ages of the same dietary treatment. * denotes significant differences between dietary treatments at the same age. Significance was set at $P < 0.05$.

Additional References

1. Kuznetsov, A.V; Veksler, V.; Gellerich, F.N.; Saks, V.; Margreiter, R.; Kunz, W.S. Analysis of mitochondrial function in situ in permeabilized muscle fibers, tissues and cells. *Nat. Protoc.* **2008**, *3*, 965–976, doi:10.1038/nprot.2008.61.
2. Larsen, S.; Nielsen, J.; Hansen, C.N.; Nielsen, L.B.; Wibrand, F.; Stride, N.; Schroder, H.D.; Boushel, R.; Helge, J.W.; Dela, F.; et al. Biomarkers of mitochondrial content in skeletal muscle of healthy young human subjects. *J. Physiol.* **2012**, *590*, 3349–3360, doi:10.1113/jphysiol.2012.230185.
